# A randomized controlled double-blinded prospective study of the efficacy of clonidine added to bupivacaine as compared with bupivacaine alone used in supraclavicular brachial plexus block for upper limb surgeries

**DOI:** 10.4103/0019-5049.72646

**Published:** 2010

**Authors:** Shivinder Singh, Amitabh Aggarwal

**Affiliations:** Department of Anaesthesiology and Critical Care, Armed Forces Medical College, Pune, India

**Keywords:** Anaesthetic techniques, brachial plexus, bupivacaine, clonidine, pharmacology, regional

## Abstract

We compared the effects of clonidine added to bupivacaine with bupivacaine alone on supraclavicular brachial plexus block and observed the side-effects of both the groups. In this prospective, randomized, double-blinded, controlled trial, two groups of 25 patients each were investigated using (i) 40 ml of bupivacaine 0.25% plus 0.150 mg of clonidine and (ii) 40 ml of bupivacaine 0.25% plus 1 ml of NaCl 0.9, respectively. The onset of motor and sensory block and duration of sensory block were recorded along with monitoring of heart rate, non-invasive blood pressure, oxygen saturation and sedation. It was observed that addition of clonidine to bupivacaine resulted in faster onset of sensory block, longer duration of analgesia (as assessed by visual analogue score), prolongation of the motor block (as assessed by modified Lovett Rating Scale), prolongation of the duration of recovery of sensation and no association with any haemodynamic changes (heart rate and blood pressure), sedation or any other adverse effects. These findings suggest that clonidine added to bupivacaine is an attractive option for improving the quality and duration of supraclavicular brachial plexus block in upper limb surgeries.

## INTRODUCTION

The supraclavicular brachial plexus block provides anaesthesia of the entire upper extremity in the most consistent and time-efficient manner.

Since the ‘80s, clonidine has been used as an adjunct to local anaesthetic agents in various regional techniques to extend the duration of block. The results of previous studies on the usefulness of clonidine on brachial plexus block have been mixed. Some studies have shown that clonidine prolongs the effects of local anaesthetics,[1–3] but other studies have failed to show any effect of clonidine, independently from the type of local anaesthetic used (ropivacaine, bupivacaine and mepivacaine).[4–7] Moreover, others have indicated an increased incidence of adverse effects like sedation, hypotension and bradycardia.[4,6–9] Clonidine has been shown to be of benefit for use in central neuraxial blocks and other regional blocks by increasing the duration and intensity of pain relief[10–12] as also by decreasing the systemic and local inflammatory stress response.[13,14] Also, there is no reason for it to be ineffective, specifically in brachial plexus blocks. This randomized, double-blind and placebo-controlled study tested the hypothesis that inclusion of clonidine with the local anaesthetic prolongs the duration of analgesia in supraclavicular brachial plexus block.

## METHODS

The study protocol of this prospective, randomized, double-blinded, placebo-controlled trial was approved by the Hospital Ethics Committee. All participants gave written informed consent. Fifty patients, ASA physical status I–III, 18 years of age or older, undergoing surgery of the upper limb, were recruited. Excluded from the study were patients for whom supraclavicular brachial plexus block or the study medications were contraindicated or those who had a history of significant neurological, psychiatric, neuromuscular, cardiovascular, pulmonary, renal or hepatic disease or alcohol or drug abuse, as well as pregnant or lactating women. Also barred from the study were patients taking medications with psychotropic or adrenergic activities and patients receiving chronic analgesic therapy. Pre-medication was given with tablet Alprazolam 0.25 mg orally at 22:00 h on the night before surgery and at 06:00 h on the morning of the surgery. No additional sedative medication was administered in the first 60 min after injection of the study dose.

In our study, two groups (*n*=25) were investigated: Group I (bupivacaine–clonidine) received 40 ml of bupivacaine 0.25% plus 0.150 mg of clonidine and Group II (bupivacaine) received 40 ml of bupivacaine 0.25% plus 1 ml of NaCl 0.9%. The anaesthetic solution was prepared according to a random-number table by means of a computer-generated randomization list by an anaesthetist not otherwise involved in the study. The anaesthetist performing the block was blinded to the treatment group. All observations were carried out by a single investigator who was also blinded to the treatment group.

Patients’ pulse rate, electrocardiogram and non-invasive blood pressure were recorded and a wide bore intravenous line was established. The patients were administered a brachial plexus block by supraclavicular approach. The site of injection was shaved and disinfected. The injection site was infiltrated with 1 ml of lidocaine 2% subcutaneously. A nerve stimulator (Stimuplex Dig RC; Braun Melsungen AG, Germany) was used to locate the brachial plexus. The location end point was a distal motor response with an output lower than 0.6 mA. During injection, negative aspiration was performed every 6.5–7.0 ml to avoid intravascular injection. Plexus block was considered successful when at least two out of four nerve territories (ulnar, radial, median and musculocutaneous) were effectively blocked.

Sensory and motor block of the musculocutaneous, radial, ulnar and median nerve were determined immediately and at 5, 10, 30, 60, 120, 180, 240, 360 and 480 min after completion of the injection. Patients were asked to note complete recovery of sensation, which was then verified by an anaesthetist or a nurse.

Sensory block was determined by the response to pin prick using a visual analogue scale (VAS) from 100 [no sign of sensory block (maximal pain)] to 0 [complete sensory block (no pain)]. Sensory onset of each nerve was assessed by the pin prick method.

Motor block was determined according to a modified Lovett rating scale, ranging from 6 (usual muscular force) to 0 (complete paralysis) as follows: thumb abduction for the radial nerve, thumb adduction for the ulnar nerve, thumb opposition for the median nerve and flexion of elbow for the musculocutaneous nerve.

The duration of sensory block was defined as the time interval between injection and complete recovery of sensation.

Also measured at the above-mentioned time points were heart rate, non-invasive blood pressure, oxygen saturation and sedation. The sedation score ranged from 1 (alert) to 4 (asleep, not arousable by verbal contact).

Patients were observed for any discomfort, nausea, vomiting, shivering, bradycardia, pain and any other side-effects. Any need for additional medication was noted. Blood loss during surgery was calculated by the gravimetric method with a view to replace the blood loss if it was more than the maximum allowable blood loss.

Results were expressed as mean±SD (SEM). Demographic and haemodynamic data were subjected to statistical analysis by using two sample *t*-tests. For statistical analysis of modified Lovett rating scale, VAS and sedation score, not normally distributed, a non-parametric test “Wilcoxon Mann Whitney test” was applied. The time of recovery of sensation and adverse effects were analyzed by the Chi square test/Fisher’s exact test. A *P*-value <0.05 was considered statistically significant. Taking α=0.05 to detect difference in recovery of sensation at 8 h in the two groups as 56% and taking sample size of 25 in each group, the power of the study is approximately 75%.

## RESULTS

### Demographic data

There were no differences between the clonidine and the control groups regarding age, sex, weight and height [Table 1] or the site of surgery.

**Table 1:** Demographic data

	Group I (*n*=25)	Group II (*n*=25)
Sex (M/F)	7 (28%)/18 (72%)	4 (16%)/21 (84%)
Age (years)	36.04±10.43	33.68±7.83
Height (cm)	163.8±3.42	162.08±3.83
Weight (kg)	62.76±4.10	62.08±3.25

### Comparison of modified lovett rating scale

**Figure 1:**
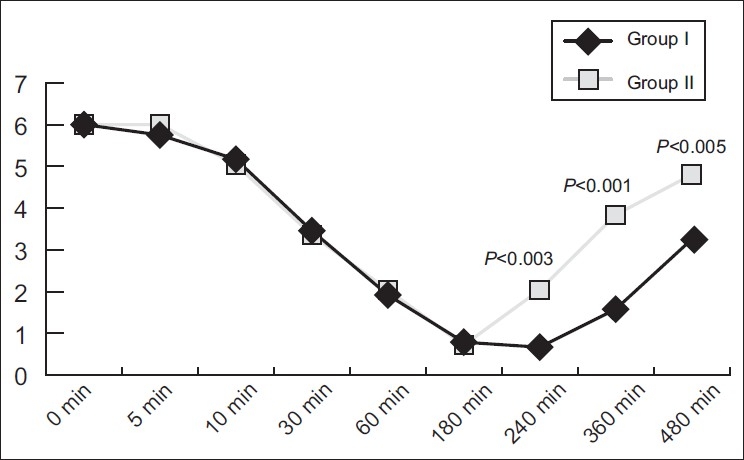
Comparison of the modified Lovett rating scale

The modified Lovett rating scale at baseline and intra-operatively was comparable in both the clonidine and the control group. However, post-operatively, after 240 min, the modified Lovett rating scale was lower in the clonidine group when compared with the control group (0.67±1.61 vs. 2.04±1.67), and it was statistically significant (*P*<0.05) [Figure 1].

### Comparison of VAS

The VAS of the two groups [Table 2] was consistently lower at all times in the clonidine group during onset till 30 min. From 30 to 240 min, when there was an intense block in both the groups, the VAS score was 0, after which, in the control group, it started rising while remaining low in the clonidine group. Because the VAS score was significantly less from 5 to 30 min (*P*-value at 5 min 0.043, at 10 min 0.008 and at 30 min 0.007), we concluded that onset with clonidine was faster. Again, after 240 min, the VAS was significantly lower and thus we concluded that the action was prolonged.

**Table 2:** Comparison of visual analogue score

	Baseline	5 min	10 min	30 min	60 min	180 min	240 min	360 min	480 min
Group I mean±SD	100±0.00	43.60±22.15	20.0±19.58	2.80±6.78	0.80±4.00	0.00±0.0	0.00±0.00	0.00±0.0	0.00±0.0
Group II mean±SD	100±0.00	55.20±15.31	33.60±13.50	9.60±10.99	2.80±5.42	0.00±0.0	0.80±4.00	6.80±8.52	19.60±17.19
*P*-value	1.00	0.043	**0.008**	**0.007**	0.053	1.00	0.317	**0.001**	**0.001**

### Time to recovery of sensation

There was no recovery of sensation in both groups up to 2 h. From 2 to 4 h, 28% of the patients of the control group had recovery of sensation while none of the patients of the clonidine group had recovery of sensation. The difference was statistically significant (*P*<0.05).

**Figure 2:**
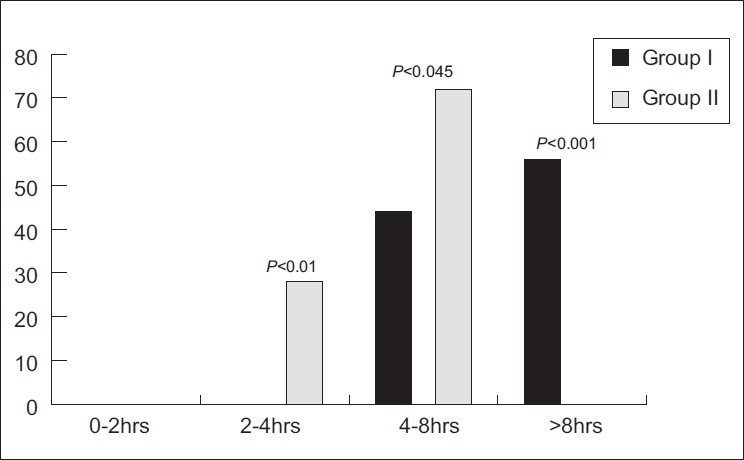
Time of recovery of sensation

Between 4 and 8 h, 72% of the patients of the control group had recovery of sensation as compared with 44% of the patients of the clonidine group, the comparison being statistically significant (*P*<0.05) [Figure 2]. In a majority of the patients (56%) of the clonidine group, recovery of sensation occurred after 8 h whereas in the control group, all patients had recovered sensations by 8 h, and the difference was statistically significant (*P*<0.05) [Table 3], showing a prolongation of block in the clonidine group.

**Table 3:** Recovery of sensation before and after 8 h

Time of recovery of sensation	Group I (*n*=25)	Group II (*n*=25)
<8 h	11 (44)	25 (100)
>8 h	14 (56)	0

*P*-value=0.0000047, Figures in parentheses are in percentages

### Comparison of sedation score

The sedation score between the clonidine and the control group was comparable throughout the study period. All the patients were alert (sedation score=1) in both groups at all times of observation.

### Comparison of saturation of oxygen

The saturation of oxygen between the clonidine and the control group was comparable throughout the study period. All the patients had saturation of oxygen >99% in both groups at all times of the observation.

### Comparison of heart rate

The baseline heart rate was lower in the clonidine group than in the control group. The perioperative and post-operative heart rate was variable at each time interval and was also lower in the clonidine group in comparison with the control group; however, the difference was not significant (*P*>0.05).

### Comparison of blood pressure

The baseline blood pressure was comparable in both the clonidine and the control group. The maximum fall in systolic and diastolic blood pressures in the clonidine group was noted at 60 min. However, in the control group, this was observed at 10 min for systolic and 30 min for diastolic blood pressures, respectively. The peri- and post-operative blood pressure was variable at each time interval in both groups and was statistically insignificant (*P*>0.05).

### Side-effects

No side-effects were observed in both the clonidine and the control group throughout the study period.

## DISCUSSION

Supraclavicular blocks are performed at the level of the brachial plexus trunks. Here, almost the entire sensory, motor and sympathetic innervations of the upper extremity are carried in just three nerve structures (trunks), confined to a very small surface area. Consequently, typical features of this block include rapid onset, predictable and dense anaesthesia along with its high success rate.

Clonidine and local anaesthetic agents have a synergistic action. Clonidine enhances both sensory and motor blockade of neuraxial and peripheral nerves after injection of local anaesthetic solution, without affecting the onset.[10–12] This is thought to be due to blockage of conduction of A delta and C fibres, increase in the potassium conductance in isolated neurons *in vitro* and intensification of conduction block achieved by local anaesthetics.

We found a significant difference in the onset of sensory block (as assessed by VAS) between the two groups. The VAS of the two groups was comparable at baseline. Thereafter, the VAS scale was lower in the clonidine group than in the control group (43.60±22.15 vs. 55.20±15.31) up to 180 min. At 360 and 480 min, the VAS score was again lower in the clonidine group (0.00±0.00 vs. 6.80±8.52), and this was statistically significant (*P*<0.05). These findings indicate faster onset of sensory block and prolongation of analgesia with use of clonidine. Most authors have reported no effect on the onset of block, which is at variance with our results,[12] This needs further evaluation. However, the prolongation of analgesia observed is consistent with other trials performed at the brachial plexus,[1–3] popliteal block[15] and in another study in children undergoing a variety of blocks, which demonstrated that the addition of clonidine to bupivacaine and ropivacaine can extend sensory block by a few hours and increase the incidence of motor blocks.[16]

Among the studies showing no positive effect[4–6] of clonidine as an additive to brachial plexus blocks, various discrepancies have been discussed.[16] In one, patients were not followed long enough (12 h) before any effect of clonidine could be detected.[6]

In another study, the authors found (surprisingly) that the time to first administration of opioids after the nerve block was shorter in patients who received local anaesthetic and clonidine compared with those who received local anaesthetic only.[4]

The modified Lovett rating scale at baseline and intraoperatively was comparable in both the groups. However, post-operatively, after 240 min, the modified Lovett rating scale was significantly lower (*P*<0.05) in the clonidine group (0.67±1.61 vs. 2.04±1.67). Patients in the control group had a recovery of sensations within 8 h whereas only 56% of the patients of the clonidine group had a recovery of sensation after 8 h, this too being clinically highly significant (*P*-value <0.001).

Thus, it is evident that the recovery of sensation was prolonged in the clonidine group. Our result concurs with other similar studies.[9,17,18] Thus, we favor the hypothesis that clonidine exerts an effect directly on the nerve fibre as a result of a complex interaction between clonidine and axonal ionotropic, metabolic or structure proteins (=receptors), which was shown in different laboratory studies.[19,20]

We also found an enhancement of perioperative analgesia and prolongation of recovery of sensation in the clonidine group, well beyond the pharmacological effect of either clonidine or bupivacaine. Direct modulation of the activity of sensory nerve fibres could conceivably explain the difference between the two groups in our study. Alternatively, this could have been a result of an overall better quality of anaesthesia at all times of surgery. Regardless of the mechanism, clonidine was found to have a valuable advantage in the field of peripheral nerve blocks when added to bupivacaine.

The difference in perioperative heart rate, blood pressure, sedation scores and oxygen saturation in both the groups was statistically insignificant (*P*>0.05).

The results of our study showed stable perioperative haemodynamics with the use of clonidine. Moreover, sedation, which is often associated with the use of clonidine,[17,18] was not apparent in our study.

Most of the studies conducted using clonidine in regional anaesthesia did not report any adverse effects.[9] However, studies by Buttner *et al*. and Bernard *et al*. reported the incidence of hypotension and bradycardia with the use of clonidine.[18,21] In our study, no side-effects were observed in both the clonidine and the control group throughout the study period.

To summarize, our study suggests that clonidine 0.150 mg in 40 ml of 0.25% bupivacaine significantly enhances the quality of supraclavicular brachial plexus block in upper limb surgeries by a faster onset and prolonged duration of sensory and motor block, enhancing post-operative analgesia. These benefits are not associated with any haemodynamic changes, sedation or other adverse effects.

In conclusion, clonidine added to bupivacaine is an attractive option for improving the quality and duration of supraclavicular brachial plexus block in upper limb surgeries.
